# Supplementary material for: Correlative cryo-electron microscopy reveals the structure of TNTs in neuronal cells
Source: Nat Commun. 2019 Jan 21;10:342. doi: 10.1038/s41467-018-08178-7 (PMC6341166; doi:10.1038/s41467-018-08178-7)
Supplement: Supplementary file 1 — Supplementary Information [file 41467_2018_8178_MOESM1_ESM.pdf]

Supplementary Material

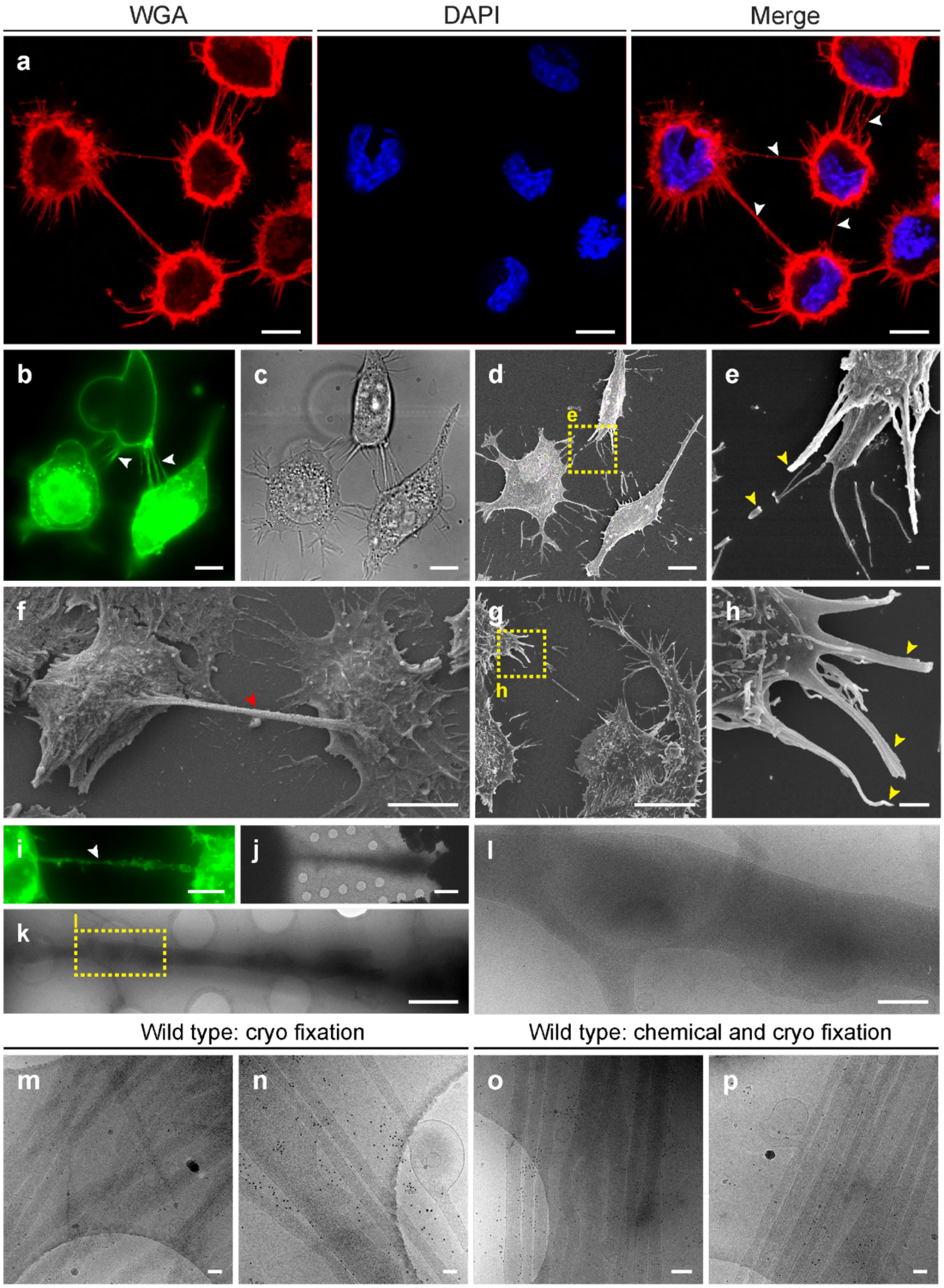

Supplementary Figure 1. Morphology of TNTs in CAD Cells using Fluorescence, Scanning-, and Cryo- Electron Microscopy

(**a**) Representative confocal micrographs displaying CAD cells stained with WGA (red) to label the plasma membrane and TNTs, and DAPI (blue) to label the nuclei. CAD cells connected via TNTs stained with WGA (green) and imaged by fluorescence (**b**) and phase contrast (**c**) microscopy. (**d**–**e**) Ultrastructure of CAD cells connected by TNTs in **b**–**c** analyzed by SEM at low- (**d**) and high- (**e**, yellow dashed box in **d**) magnification reveals that TNTs break during EM sample preparation. (**f**) SEM micrograph of CAD cells reveals that only thick TNTs endure EM sample preparation. (**g**–**h**) Representative SEM micrographs of CAD cells obtained at low- (**g**) and high- (**h**, yellow dashed box in **g**) magnification reveals that broken TNTs are comprised of single cylindrical units bundled together. (**i**–**l**) TNTs in CAD cells imaged by fluorescence (**i**), low- (**j**), intermediate (**k**), and high- (**l**, yellow dashed boxes in **k**) magnification cryo-TEM images reveal single, thick TNTs connecting cells. White arrowhead shows TNTs connecting cells by fluorescence microscopy. (**m**–**p**) Representative electron micrographs displaying iTNTs connecting CAD cells fixed by cryo fixation (rapid freezing) (**m**–**n**), and chemical fixation **(o**–**p**), show no differences in structural characteristics, in particular, the presence of parallel actin filaments and vesicular compartments within iTNTs. White arrowheads show TNTs connecting cells by confocal (**a**) and fluorescence microscopy (**b**, **i**)**.** Yellow arrowheads show thin TNTs that break during EM sample preparation (**e**, **h**)**.** Red arrowhead shows thick TNTs that endure EM sample preparation (**f**). Scale bars: **a**–**c**, **f**–**g**, 10 µm**; d**, **e**, **i**–**j,** 500 nm; **h**, 1 µm; **k**, 2 µm; **m**–**p**, 100 nm.

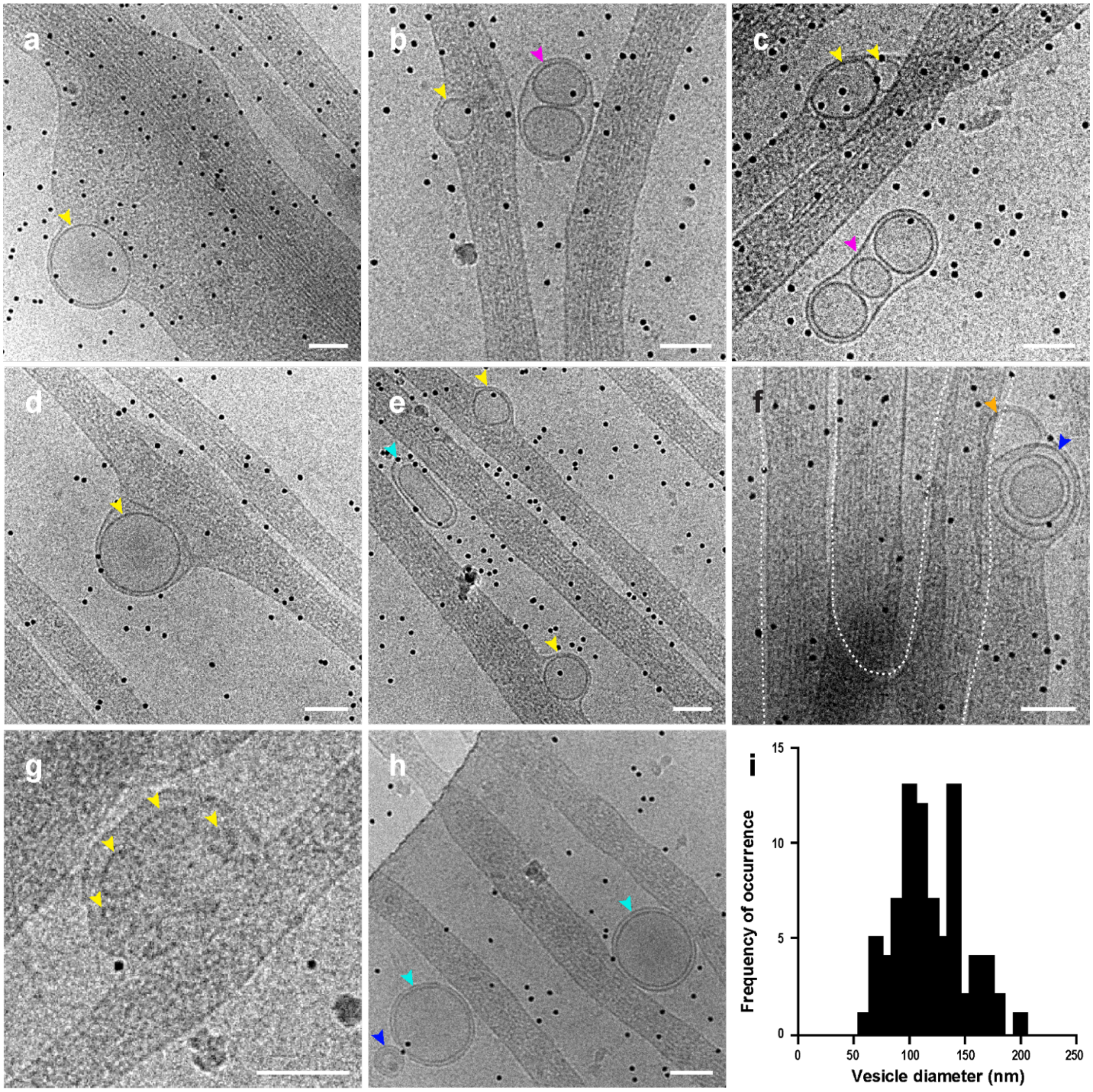

Supplementary Figure 2. Gallery of Different Membrane Compartments Observed Outside and Within iTNTs

(**a–h**) Representative cryo-electron micrographs of membrane compartments and vesicular structures associated with iTNTs. Blue arrowheads in **f** and **h** indicate vesicles with a multi-lamellar appearance. Yellow arrowheads in **a**–**g** show single vesicular compartments. Turquoise arrowheads in **e**–**h** show extracellular vesicles with a double membrane. Pink arrowheads in **b**–**c** show groups of vesicles surrounded by a secondary membrane. Vesicles squeeze actin bundles together and create a bump on the plasma membrane (**a**–**g**). (**i**) Distribution of the diameter of vesicles in nm. Scale bars: **a–h**, 100nm.

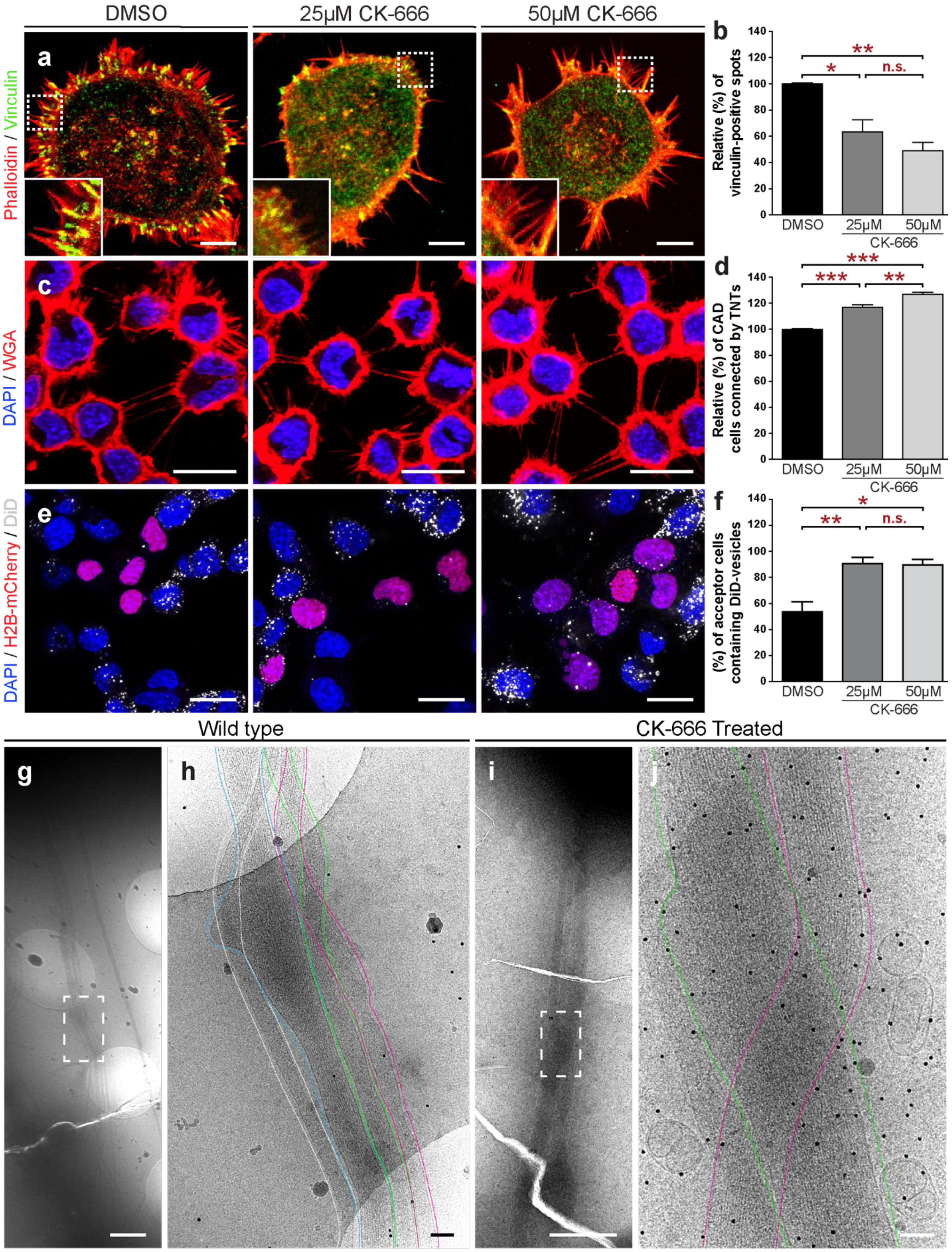
DMSO
25μM CK-666
50μM CK-666
a
Phalloidin / Vinculin
b
Relative (%) of vinculin-positive spots
**
*
n.s.
DMSO
25μM
50μM
CK-666
c
DAPI / WGA
d
Relative (%) of CAD cells connected by TNTs
***
***
**
e
DAPI / H2B-mCherry / DiD
f
(%) of acceptor cells containing DiD-vesicles
*
**
n.s.
Wild type
CK-666 Treated
g
h
i
j

Supplementary Figure 3. Effect of Arp 2/3 Complex Inhibition on TNTs

(**a**) Representative confocal micrographs displaying CAD cells immunostained with anti-vinculin (green) to label peripheral focal adhesions, and phalloidin (red) to label actin. (**b**) Statistical analysis of vinculin positive spots after treatment with DMSO and CK-666 (25 μM and 50 μM), respectively. Only peripheral focal adherent spots located at the base of the protrusion (insets, a), and found in low focal planes of the projection, were quantified as adherent filopodia. (**c**) Confocal micrographs showing cells stained with DAPI (blue) and WGA (red) to label the plasma membrane and TNTs. (**d**) Statistical analysis showing the relative percentage of cells connected by TNTs. TNTs were detected in high focal planes of the projection. (**e**) Confocal micrographs of the co-culture between cells stained with DAPI (donor and acceptor) (blue), WGA (donor and acceptor) (not shown), DiD (donor) (grey), and transfected with H2B-mCherry (acceptor cells) (red). (**f**) Statistical analysis showing the percentage of acceptor cells containing DiD-labeled vesicles transferred from donor cells. (**g**–**j**) Representative electron micrographs show wild-type (**g**–**h**) and CK-666 treated (i–j) iTNTs connecting CAD cells. (**g**, **i**) Low magnification cryo-TEM images of TNTs. (**h**, **j**) High-magnification electron micrographs correspond to white dashed rectangles in **g** and **i**, respectively. (**g**–**j**) CK-666 treated TNTs look indistinguishable from untreated controls, which display a bundled and twisted phenotype with actin arranged in a parallel fashion. The plasma membrane of iTNTs were drawn with colored dotted lines in **h** and **j** to show membrane boundaries. Scale bars: **a**, **e**, 20μm; **c**, 10μm; **g**, **i**, 1μm; **h**, **j**, 100nm. Data is represented as mean (±SEM), normalized to control cells arbitrarily set at 100% of at least 3 independent experiments. *$p < 0.05$, **$p < 0.005$, ***$p < 0.0005$, n.s. = not significant, according to one-way ANOVA test with Tukey correction.

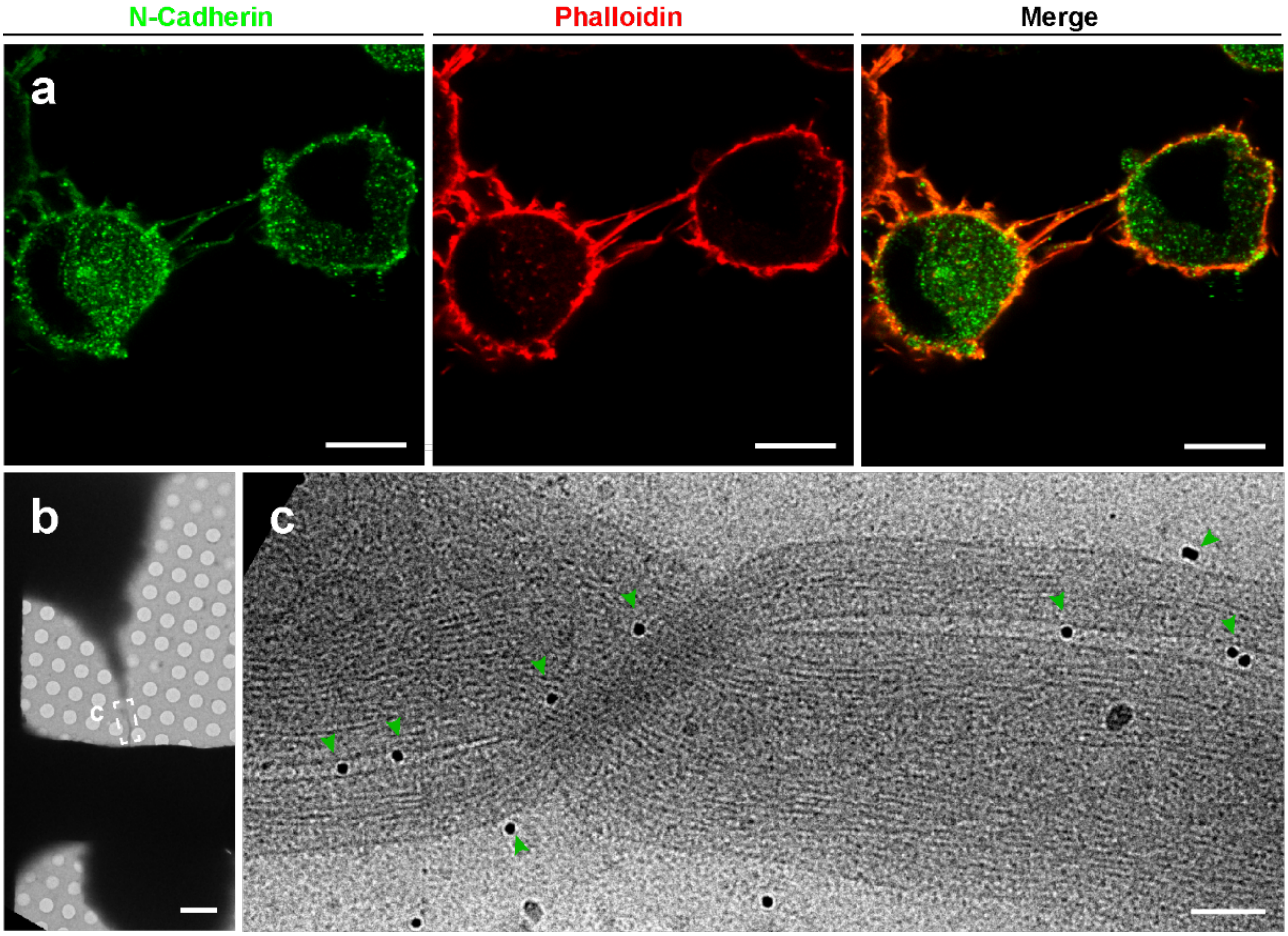

Supplementary Figure 4. N-Cadherin detection on TNTs using CLEM

(**a**) Confocal micrographs displaying CAD cells connected by TNTs labeled with an anti-N-Cadherin antibody (green) and Phalloidin (red). (**b**) low magnification electron micrograph of TNT-connected CAD cells immunolabeled with an anti-N-Cadherin primary antibody, followed by a secondary antibody coupled to 10nm gold particles. (**c**) High magnification cryo-ET slice corresponding to the white dashed rectangle in **b**. Green arrowheads indicate N-Cadherin gold particles at the surface and between iTNTs. Scale bars: **a**, 10μm; **b**, 5 μm; **c**, 100 nm.

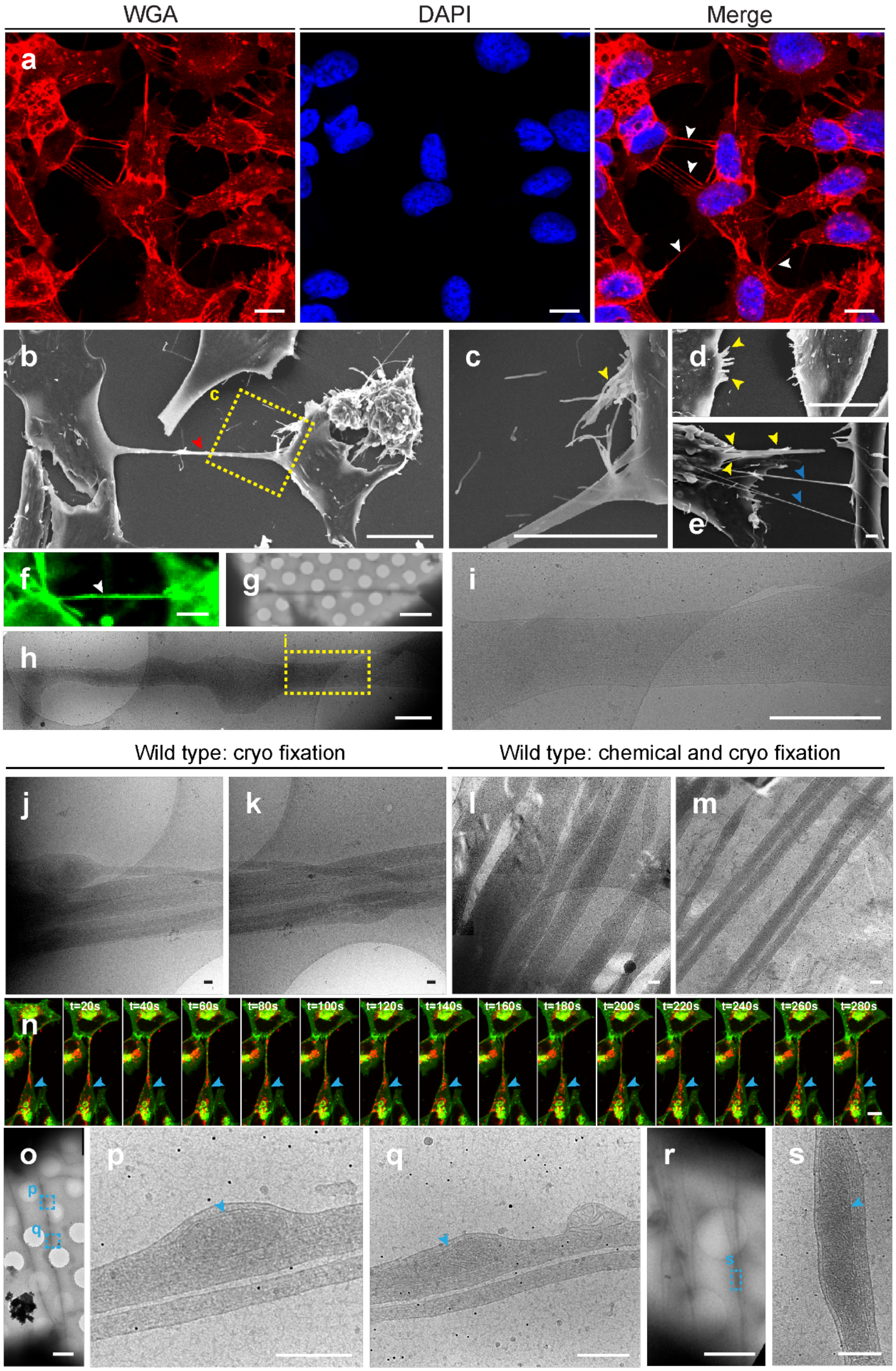

WGA
DAPI
Merge
a
b
c
d
e
f
g
h
i
Wild type: cryo fixation
Wild type: chemical and cryo fixation
j
k
l
m
n
t=20s
t=40s
t=60s
t=80s
t=100s
t=120s
t=140s
t=160s
t=180s
t=200s
t=220s
t=240s
t=260s
t=280s
o
p
q
r
s

Supplementary Figure 5. Structural and Functional Analysis of TNTs in SH-SY5Y Cells

(**a**) Representative confocal micrographs displaying SH-SY5Y cells stained with WGA (red) to label the plasma membrane and TNTs, and DAPI (blue) to label the nuclei. (**b**–**e**) SEM micrograph of SH-SY5Y cells obtained at low- **(b)** and high- (**c**, yellow dashed box in **b**) magnification reveals that single thick TNTs endure EM sample preparation, while thin ones bundled together break. White arrowheads show TNTs connecting cells by fluorescence microscopy **(a).** Yellow arrowheads show thin TNTs that break during EM sample preparation (**c**, **d**, **e**). Red arrowheads show thick TNTs that endure EM sample preparation (**b**). Blue arrowheads show an example of thin TNTs in SH-SY5Y cells enduring EM sample preparation (**e**). (**e**–i) TNTs in SH-SY5Y cells imaged by fluorescence (**f**), low- (**g**), intermediate- (**h**), and high- (**i**), yellow dashed boxes in **h** magnification cryo-TEM images reveal single, thick TNTs connecting cells. White arrowheads in **f** show TNTs connecting cells by fluorescence microscopy. (**j**–**m**) Representative electron micrographs displaying iTNTs connecting SH-SY5Y cells fixed by cryo fixation (rapid freezing) (**j**, **k**), and chemical fixation (**l**, **m**), show no differences in structural characteristics. (**n**) Time-lapse images of wild-type SH-SY5Y cells stained with WGA (green) and MitoTracker (red) show mitochondria traveling across a TNT unidirectionally and accumulating in the cytoplasm of the neighboring cell (blue arrowhead). Image intensity was increased to better visualize (thin) TNT connecting the two cells. (**o**, **r**) Low magnification cryo-TEM micrographs of SH-SY5Y cells connected by iTNTs. (**p**, **q**, **s**) High magnification cryo TEM images of TNTs shown in **o**, **r**, containing mitochondria (blue arrowheads) within tubes. Images reveal mitochondria stretched inside tubes, surrounded by actin filaments. Scale bars: **a**–**d**, **n**, 10 μm; **e**, 1 μm, **f**, **g**, 5 μm; **o**, **r**, 2 μm; **h**, **i**, 500 nm, **j–m, p, q, s,** 100 nm.

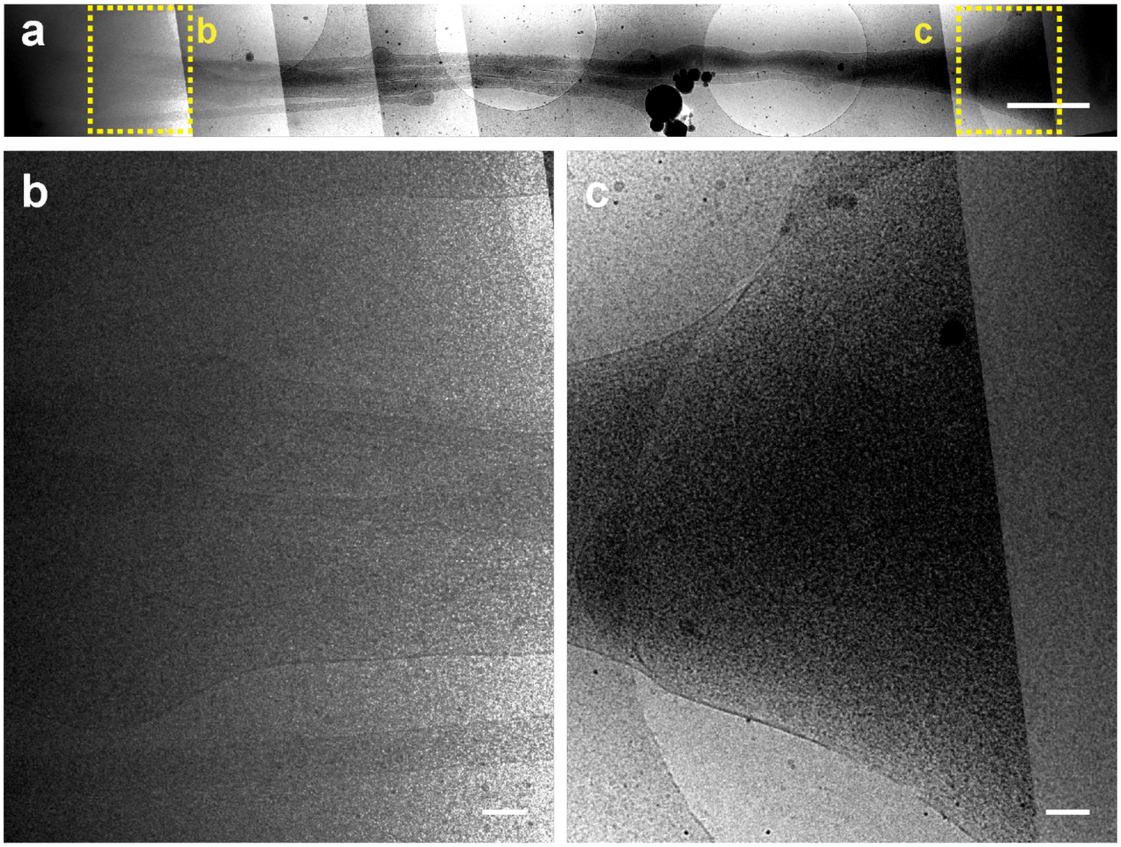

Supplementary Figure 6. iTNT Contact Sites by cryo-TEM

(**a**) Collage of high magnification cryo-TEM micrographs of SH-SY5Y cells connected by iTNTs. (**b**–**c**) High magnification cryo TEM micrographs of TNT contact zones shown in **a** (yellow dashed rectangles). Images reveal that the ice formed around the periphery of cells, including the ends of iTNTs, is too thick for the electron beam to go through. Scale bars: **a**, 1μm; **b**, **c**, 100nm.

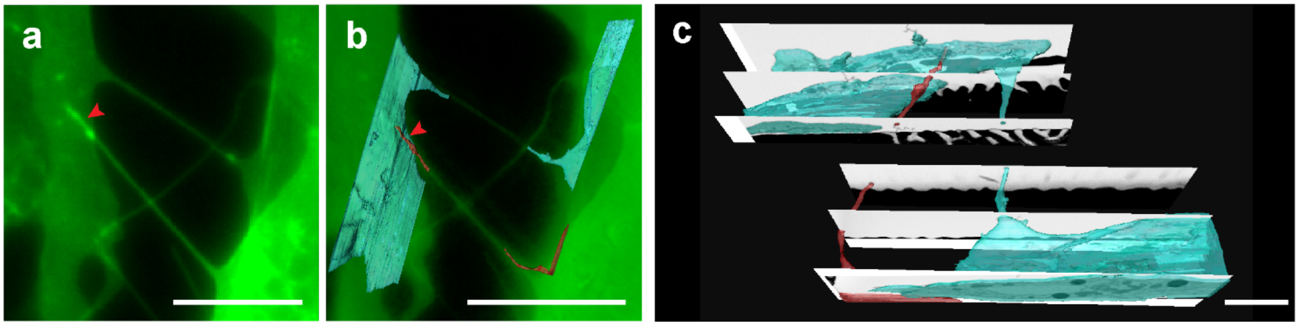

Supplementary Figure 7. FIB-SEM of TNTs in SH-SY5Y Cells Reveal Open-Ended Contact Sites (**a**) Confocal micrograph of SH-SY5Y cells stained with WGA (green). (**b**) Overlay of 3D rendering of FIB-SEM tomogram segmentations over fluorescent counterpart reveal two connections: one open-ended TNT, and one invaginating inside the cytoplasm of the opposing cell (red arrowhead). (**c**) 3D rendering of FIB-SEM tomogram segmentation shown in **b**. Scale bars: **a**–**b**, 10μm; **c**, 3μm.
