# Supplementary material for: Correlative cryo-electron microscopy reveals the structure of TNTs in neuronal cells
Source: Nat Commun. 2019 Jan 21;10:342. doi: 10.1038/s41467-018-08178-7 (PMC6341166; doi:10.1038/s41467-018-08178-7)
Supplement: Supplementary file 3 — Description of Additional Supplementary Files [file 41467_2018_8178_MOESM3_ESM.docx]

**Description of Supplementary Files**

**File Name:** Supplementary Movie 1

**Description:** Representative slices of a reconstructed tomogram displaying iTNTs connecting two CAD cells shown in Fig. 2f. Scale bar: 200nm.

**File Name:** Supplementary Movie 2

**Description:** Representative slices from the subregion of a reconstructed tomogram, displaying iTNTs connecting two CAD cells shown in Fig. 2g. These slices display several distinct features of iTNTs such as: long actin arrangements; vesicles and other vesicular compartments within iTNTs; iTNT coils; vesicle-to-actin, vesicle-to-membrane, and iTNT-to-iTNT links. Scale bar: 200nm.

**File Name:** Supplementary Movie 3

**Description:** Representative slices from the subregion of a reconstructed tomogram, displaying iTNTs connecting two CAD cells shown in Fig. 2h. Slices display membranous compartment within one of the iTNTs observed and an extracellular vesicle. Scale bar: 200nm.

**File Name:** Supplementary Movie 4

**Description:** Slices of a reconstructed tomogram displaying iTNTs connecting two CAD cells shown in Fig. 3b-3c. CADcells were immunogold labeled with an anti-N-Cadherin primary antibody and a secondary antibody coupled to a 10nm gold particle. Slices display thin threads connecting iTNTs positively labeled for N-Cadherin. Scale bar: 100nm.

**File Name:** Supplementary Movie 5

**Description:** Slices of a reconstructed tomogram displaying iTNTs connecting two CAD cells shown in Fig. 2k. Slices display four iTNTs parallel to each other, connected by long, thin threads. Scale bar: 200nm.

**File Names:** Supplementary Movies 6 - 7

**Description:** Slices of a reconstructed tomogram displaying iTNTs connecting CAD cells transfected with GFP-Myo10. Vesicular structures observed inside iTNTs correspond to GFP-Myo10 puncta by FM (Fig. 3f and 3g-3h, respectively). Scale bar: 100nm.

**File Name:** Supplementary Movie 8

**Description:** Slices of a reconstructed tomogram displaying iTNTs connecting two CAD cells shown in Fig. 4a. Scale bar: 100nm.

**File Name:** Supplementary Movie 9

**Description:** Slices of a reconstructed tomogram displaying two crossing filopodia protruding from one wild-type CAD cell. Slices display straight and branched actin configurations within filopodia. (Fig. 5b). Scale bar: 200nm.

**File Name:** Supplementary Movie 10

**Description:** Slices of a reconstructed tomogram displaying one filopodia protruding from a GFP-VASP-transfected CAD cell. (Fig. 5d). Scale bar: 100 nm.

**File Name:** Supplementary Movie 11

**Description:** Slices of a reconstructed tomogram displaying one filopodia protruding from a GFP-transfected CAD cell. (Fig. 5f). Scale bar: 100 nm.

**File Name:** Supplementary Movie 12

**Description:** Slices of a reconstructed tomogram displaying one filopodia protruding from a wild-type CAD cell. (Fig. 5h) Scale bar: 200 nm.

**File Name:** Supplementary Movie 13

**Description:** Dynamics of mitochondria labeled with Mitotracker (red) transferring unidirectionally between two SH-SY5Y cells labeled with WGA (green) via a TNT. Live imaging was acquired over 20 min at 20 sec intervals. Mitochondria puncta moved inside the TNT in a unidirectional fashion at an average velocity of 0.05 μm/sec (SD=0.03 μm/sec) (Fig. 6g). Scale bar: 20 μm.

**File Name:** Supplementary Movie 14

**Description:** Dynamics of mitochondria labeled with Mitotracker (red) transferring unidirectionally between two SH-SY5Y cells labeled with WGA (green) via a TNT and accumulating in the acceptor cell. (Supplementary Figure 6h). Scale bar: 10um.

**File Name:** Supplementary Movie 15

**Description:** Slices of a reconstructed tomogram displaying two iTNTs connecting two SH-SY5Y cells that contain a mitochondria inside. (Fig. 6m)

**File Name:** Supplementary Movie 16

**Description:** FIB-SEM tomographic volume of open-ended TNTs connecting two CAD cells, surface rendition, and manually annotated segmentation of the tomographic data created with the Amira software package. Color labels are: cyan, two cell bodies connected by three open-ended iTNTs (yellow, pink, and blue); red, two closeended protrusion stemming from opposite cells. (Fig. 7b-7d).

**File Name:** Supplementary Movie 17

**Description:** FIB-SEM tomographic volume of open-ended TNTs connecting two SH-SY5Y cells, surface rendition, and manually annotated segmentation of the tomographic data created with the Amira software package. Color labels are: cyan, two cell bodies connected by an open-ended iTNT; yellow, close-ended protrusion that splits off the iTNT in cyan; blue, close-ended protrusion that makes contact with the iTNT in cyan; red, close-ended protrusion that lays over the apposing cell body. (Fig. 7e-7f).

**File Name:** Supplementary Movie 18

**Description:** FIB-SEM tomographic volume of open- and close-ended TNTs connecting two SH-SY5Y cells and manually annotated segmentation of the tomographic data created with the Amira software package. Color labels are: cyan, two cell bodies connected by the open-ended TNT; red, cell that extends a protrusion which invigilates the opposing cell (in cyan). (Supplementary Figure 7).
