# Supplementary material for: Correlative cryo-electron microscopy reveals the structure of TNTs in neuronal cells
Source: Nat Commun. 2019 Jan 21;10:342. doi: 10.1038/s41467-018-08178-7 (PMC6341166; doi:10.1038/s41467-018-08178-7)
Supplement: Supplementary file 22 — Source Data [file 41467_2018_8178_MOESM22_ESM.docx]

| **Figure-2-Panel a** |  |
| --- | --- |
| iTNT Diameter (nm) | Frequency of occurrence |
| 0 | 0 |
| 10 | 0 |
| 20 | 0 |
| 30 | 0 |
| 40 | 1 |
| 50 | 3 |
| 60 | 12 |
| 70 | 25 |
| 80 | 43 |
| 90 | 55 |
| 100 | 54 |
| 110 | 68 |
| 120 | 70 |
| 130 | 49 |
| 140 | 36 |
| 150 | 25 |
| 160 | 21 |
| 170 | 12 |
| 180 | 15 |
| 190 | 3 |
| 200 | 2 |
| 210 | 3 |
| 220 | 2 |
| 230 | 2 |
| 240 | 0 |
| 250 | 0 |
| 260 | 1 |
| 270 | 2 |
| 280 | 1 |
| 290 | 0 |
| 300 | 1 |
| 310 | 0 |
| 320 | 1 |
| 330 | 0 |
| 340 | 0 |
| 350 | 0 |
| 360 | 0 |
| 370 | 2 |
| 380 | 0 |
| 390 | 2 |
| 400 | 1 |
| 410 | 2 |
| 420 | 0 |
| 430 | 1 |
| 440 | 1 |
| 450 | 1 |
| 460 | 1 |
| 470 | 0 |
| 480 | 0 |
| 490 | 0 |
| 500 | 1 |
| 510 | 0 |
| 520 | 0 |
| 530 | 1 |
| 540 | 1 |
| 550 | 1 |
| 560 | 0 |
| **570** | 0 |
| 580 | 0 |
| 590 | 0 |
| 600 | 0 |
| 610 | 0 |
| 620 | 0 |
| 630 | 0 |
| 640 | 0 |
| 650 | 0 |
| 660 | 0 |
| 670 | 0 |
| 680 | 0 |

| **Figure 2-Panel b** |  |
| --- | --- |
| iTNT-iTNT Surfance Distance (nm) | Frequency of occurrence |
| 0 | 0 |
| 10 | 5 |
| 20 | 57 |
| 30 | 38 |
| 40 | 39 |
| 50 | 27 |
| 60 | 16 |
| 70 | 2 |
| 80 | 5 |
| 90 | 2 |
| 100 | 1 |
| 110 | 1 |
| 120 | 2 |
| 130 | 1 |
| 140 | 3 |
| 150 | 1 |
| 160 | 2 |
| 170 | 0 |
| 180 | 0 |
| 190 | 0 |
| 200 | 1 |
| 210 | 0 |

| **Figure 6-Panel f** |  |
| --- | --- |
| **iTNT-diameter(nm)** | **Frequency of occurrence** |
| 0 | 0 |
| 10 | 0 |
| 20 | 0 |
| 30 | 0 |
| 40 | 10 |
| 50 | 28 |
| 60 | 33 |
| 70 | 34 |
| 80 | 27 |
| 90 | 32 |
| 100 | 39 |
| 110 | 51 |
| 120 | 38 |
| 130 | 37 |
| 140 | 28 |
| 150 | 25 |
| 160 | 13 |
| 170 | 10 |
| 180 | 7 |
| 190 | 6 |
| 200 | 3 |
| 210 | 6 |
| 220 | 3 |
| 230 | 1 |
| 240 | 3 |
| 250 | 1 |
| 260 | 2 |
| 270 | 6 |
| 280 | 1 |
| 290 | 2 |
| 300 | 2 |
| 310 | 3 |
| 320 | 3 |
| 330 | 1 |
| 340 | 3 |
| 350 | 0 |
| 360 | 2 |
| 370 | 4 |
| 380 | 0 |
| 390 | 1 |
| 400 | 0 |
| 410 | 0 |
| 420 | 1 |
| 430 | 0 |
| 440 | 0 |
| 450 | 1 |
| 460 | 0 |
| 470 | 0 |
| 480 | 0 |
| 490 | 1 |
| 500 | 0 |
| 510 | 0 |
| 520 | 0 |
| 530 | 0 |
| 540 | 0 |
| 550 | 0 |
| 560 | 0 |
| 570 | 0 |
| 580 | 0 |
| 590 | 0 |
| 600 | 0 |
| 610 | 0 |
| 620 | 0 |
| 630 | 0 |
| 640 | 0 |
| 650 | 0 |
| 660 | 0 |
| 670 | 0 |
| 680 | 0 |

| **Figure 6-Panel g** |  |
| --- | --- |
| **vesicles diameter (nm)** | **Frequency of occurrence** |
| 0 | 0 |
| 10 | 0 |
| 20 | 0 |
| 30 | 0 |
| 40 | 0 |
| 50 | 0 |
| 60 | 1 |
| 70 | 2 |
| 80 | 4 |
| 90 | 4 |
| 100 | 2 |
| 110 | 2 |
| 120 | 5 |
| 130 | 4 |
| 140 | 5 |
| 150 | 3 |
| 160 | 2 |
| 170 | 4 |
| 180 | 1 |
| 190 | 3 |
| 200 | 5 |
| 210 | 3 |
| 220 | 4 |
| 230 | 0 |
| 240 | 1 |
| 250 | 0 |
| 260 | 0 |
| 270 | 1 |
| 280 | 0 |
| 290 | 1 |
| 300 | 2 |
| 310 | 1 |
| 320 | 1 |
| 330 | 0 |
| 340 | 3 |
| 350 | 1 |
| 360 | 0 |
| 370 | 0 |
| 380 | 0 |
| 390 | 0 |
| 400 | 1 |
| 410 | 0 |
| 420 | 0 |
| 430 | 0 |
| 440 | 0 |

| **Supplementary Figure 2-**  **Panel i** |  |
| --- | --- |
| Vesicles Diameter (nm) | Frequency of occurrence |
| 0 | 0 |
| 10 | 0 |
| 20 | 0 |
| 30 | 0 |
| 40 | 0 |
| 50 | 0 |
| 60 | 1 |
| 70 | 5 |
| 80 | 4 |
| 90 | 7 |
| 100 | 13 |
| 110 | 12 |
| 120 | 7 |
| 130 | 5 |
| 140 | 13 |
| 150 | 2 |
| 160 | 4 |
| 170 | 4 |
| 180 | 2 |
| 190 | 0 |
| 200 | 1 |
| 210 | 0 |

**Supplementary Figure 3**

|  |  |  |  |  |  |  |  |  |  |  |  |  |  |  |
| --- | --- | --- | --- | --- | --- | --- | --- | --- | --- | --- | --- | --- | --- | --- |
| **Relative percent of TNT-connected cells** | | |  | **Relative percent of vinculin-positive spots** | | |  | **Relative percentage of # DiD vesicles/cell** | | |  | **(%) of acceptor cells containing DiD-vesicles** | | |
|  |  |  |  |  |  |  |  |  |  |  |  |  |  |  |
|  |  |  |  |  |  |  |  |  |  |  |  |  |  |  |
|  |  |  |  |  |  |  |  |  |  |  |  |  |  |  |
| **dmso** | **25** | **50** |  | **dmso** | **25** | **50** |  | **dmso** | **25** | **50** |  | **dmso** | **25** | **50** |
| 100 | 114.9 | 124.19 |  | 51.27 | 39.75 | 31.5 |  | 1.60% | 8.80% | 7.90% |  | 37 | 93 | 81 |
| 100 | 120.23 | 128.33 |  | 67 | 44.66 | 30.5 |  | 3% | 13% | 15.50% |  | 60 | 95 | 95 |
| 100 | 115.92 | 128.52 |  | 65.3 | 29.9 | 26.31 |  | 2% | 9.56% | 10% |  | 60 | 79 | 89 |
|  |  |  |  |  |  |  |  |  |  |  |  |  |  |  |
| 100.00% | 114.90% | 124.19% |  | 100.00% | 77.53% | 61.44% |  | 100.00% | 550.00% | 493.75% |  | 100.00% | 251.35% | 218.92% |
| 100.00% | 120.23% | 128.33% |  | 100.00% | 66.66% | 45.52% |  | 100.00% | 433.33% | 516.67% |  | 100.00% | 158.33% | 158.33% |
| 100.00% | 115.92% | 128.52% |  | 100.00% | 45.79% | 40.29% |  | 100.00% | 478.00% | 500.00% |  | 100.00% | 131.67% | 148.33% |
|  |  |  |  |  |  |  |  |  |  |  |  |  |  |  |
| 100.00% | 117.02% | 127.01% |  | 100.00% | 63.33% | 49.08% |  | 100.00% | 487.11% | 503.47% |  | 100.00% | 180.45% | 175.20% |
